# The Efficacy of Functional Endoscopic Sinus Surgery Combined With Triamcinolone Acetonide Aqueous Nasal Spray for the Treatment of Chronic Rhinosinusitis

**DOI:** 10.3389/fsurg.2022.855618

**Published:** 2022-05-27

**Authors:** Zhongping Hao, Huaiyu Gu, Wen Li

**Affiliations:** Department of Otolaryngology, Suzhou Hospital of Anhui Medical University, Anhui, China

**Keywords:** chronic rhinosinusitis, functional endoscopic sinus surgery, triamcinolone acetonide aqueous nasal spray, inflammatory cytokine, olfactory function

## Abstract

**Objective:**

We aimed to investigate the efficacy of functional endoscopic sinus surgery (FESS) combined with triamcinolone acetonide aqueous nasal spray (TAA AQ) for the treatment of chronic rhinosinusitis.

**Methods:**

From December 2019 to June 2021, 109 patients with chronic rhinosinusitis were classified into a control group (*n* = 50) and an experimental group (*n* = 59) according to the method of treatment. Subjects in the control group were treated with FESS while those in the experimental group were treated with FESS + TAA AQ. We then compared clinical indices, total effective rate, and the clinical symptoms of patients between the two groups. The pre- and postoperative serum levels of inflammatory cytokines were also determined. Before and 12 months after surgery, we analyzed the recovery of the nasal mucosa, olfactory function, and mucociliary transport rate of each patient. Postoperative complications were observed and recorded and the quality-of-life 12 months after surgery was ascertained.

**Results:**

Clinical indices and total effective rate were higher in the experimental group. After treatment, the VAS score and serum levels of inflammatory cytokines in the two groups both decreased, although the experimental group had lower VAS scores and inflammatory cytokine levels. Six months after treatment, olfactory function, and the recovery of nasal mucosa were improved, MTR had increased, and the total incidence of complications had reduced in the experimental group when compared with the control group. No significant difference was found between the two groups in terms of quality-of-life (*P* > 0.05).

**Conclusion:**

The combination of FESS and TAA AQ exerted a certain therapeutic effect on chronic rhinosinusitis.

## Introduction

As an inflammatory condition in the paranasal sinus mucosa, chronic rhinosinusitis (CRS) is characterized by a range of symptoms lasting for at least 12 weeks, including olfactory dysfunction, facial pain/pressure, nasal obstruction, and anterior/posterior discharge (1). CRS is common and the incidence was previously estimated to be approximately 11%; this condition also exerts a significant effect on a patient's quality-of-life and creates a significant socioeconomic burden (2). Endoscopic evidence of edema and/or pus and computed tomography (CT) scans can facilitate the diagnosis of this disease. The normal physiological function of the sinuses depends on the patency of the osteomeatal unit, a normal quantity and quality of secretions, and normal mucociliary transport (3). Patients with CRS are primarily treated by medical therapy in the form of topical nasal steroids and antibiotics. Over recent decades, the surgical management of CRS has changed due to technological improvements in endoscopy apparatus and the increased awareness of the significance of mucociliary flow and ventilation *via* the anatomical ostia for normal sinus function (4).

Functional endoscopic sinus surgery (FESS) is the gold standard in the surgical management of CRS. The emphasis of FESS is on clearing obstructions in the common drainage pathway; this restores functionality by allowing mucociliary clearance to normalize, thus improving ventilation (5). FESS has been demonstrated to be successful and leads to a significant improvement in the quality-of-life (6). However, the positive post-surgical effects are not notable until 3–4 weeks after treatment, largely due to the discomfort produced by nasal secretions, extensive crust, and mucosal edema (7). Therefore, there is a clear need to identify agents that are able to promote the clearance of the nasal cavity, the postoperative regeneration of the nasal mucosa, and novel strategies for CRS therapy. Triamcinolone acetonide aqueous nasal spray (TAA AQ) is currently indicated for the treatment of nasal symptoms associated with CRS. Previous studies have demonstrated the safety, tolerability, and efficacy of triamcinolone acetonide in CRS (8–10). Moreover, it has been reported that triamcinolone, as an operative steroid-impregnated nasal packing, exerts a positive impact on postoperative endoscopic outcomes in patients with CRS with nasal polyposis (CRSwNP) undergoing FESS (11). A previous study revealed that long-term treatment with a low dose of oral macrolide clarithromycin combined with topical TAA AQ was efficacious in patients with CRS (12). Furthermore, it has been demonstrated that patients with CRS who accepted FESS and received triamcinolone at the site of surgery showed better improvement after 8 weeks when compared with those treated with normal saline (13). Compared with other types of intranasal steroids, such as fluticasone propionate and mometasone furoate, TAA AQ has been associated with significantly higher preference, more positive sensory attributes, and better-expected compliance, thus affecting adherence to treatment (14). Therefore, we selected TAA AQ for our study design. This research was performed to confirm whether the efficacy of the combination of FESS and TAA AQ in the treatment of CRS was better than that of FESS alone.

## Materials and Methods

### Study Subjects

From December 2019 to June 2021, the clinical files of 109 patients with chronic rhinosinusitis that accepted treatment in Suzhou Hospital of Anhui Medical University were retrospectively analyzed. These patients were separated into a control group and an experimental group according to the method of treatment. The inclusion criteria were as follows: patients conformed to the CRS diagnostic criteria consisting of guidelines endorsed by the American Academy of Otolaryngology (15); chief complaints of headache, nasal obstruction, and purulent nasal discharge; nasal mucosa hyperemia, edema, and mucous discharge from middle nasal meatus and olfactory cleft, as observed by nasal endoscopy; those with a complete set of clinical data. The exclusion criteria were as follows: those with nasal papilloma, acute nasosinusitis, a chronic paranasal sinus fungus disease, acute episodes of chronic rhinosinusitis, paranasal sinus malignant tumors, a history of head trauma, intracranial tumor, and surgery; immunodeficiency disease; other malignancies; a psychiatric history; a history of respiratory tract infection; those with an incomplete set of clinical data. All patients provided signed and informed consent. The study was approved by the Ethics Committee of Suzhou Hospital of Anhui Medical University.

### Method

Patients in both groups were treated with FESS. After anesthesia, the polyps in the nasal meatus and hyperplastic granulation tissues were resected and the uncinate process was excised to open the ethmoid sinus. Then, the sinus ostium was expanded and the lesion tissues in the sinus cavity and ostium were removed. Part of the middle turbinate was resected if there were severe polypoid lesions or reverse bending of the middle turbinate as this hindered drainage. The middle turbinate would be kept if there were no significant lesions or only mild polypoid lesions. Following surgery, patients were treated with nasal saline lavage to clear the nasal secretions and dried blood and thus keep the nose clean. During the perioperative period, patients were given antibiotics (Oral roxithromycin) and nasal lavage for 14 days.

Based on FESS treatment, patients in the experimental group were managed with TAA AQ. The spray was administered through nasal inhalation 1 week before surgery (once/nostril/day). One week after surgery, each nostril was given 1–2 sprays (once in the morning and once at night); the treatment lasted for 3 months.

### Index Observation

(1) The clinical indices of patients from the two groups were compared, including intraoperative blood loss, nasal mucosal recovery time, nasal ventilation time, and hospital stay.

(2) The therapeutic effect of patients from the two groups was compared: the clinical symptoms and signs disappeared, and the results of nasal endoscopy and paranasal sinus examination were negative. Effective: the symptoms and signs of patients improved significantly while a small amount of pus was observed in the nasal cavity upon nasal endoscopy, and the patients still experienced clinical symptoms, such as a running nose and headache. Ineffective: the symptoms and signs of patients did not improve significantly and even became aggravated; total effective rate = significantly effective rate + effective rate.

(3) The clinical symptoms of patients before and after treatment were evaluated using the VAS score: the symptoms include nasal obstruction, running nose, headache, and hypogeusia; A score of 0 referred to a lack of pain while a score of 10 referred to twinge.

(4) The levels of inflammatory cytokines in patients were compared: fasting venous blood was extracted from patients in the two groups before and after treatment to detect the levels of C-reactive protein (CRP), interleukin (IL)-4, IL-5, IL-6, and IL-8 using ELISAs.

(5) Nasal mucosal recovery: we observed nasal secretion, scab, nasal mucosa color, swelling, epithelial migration, polyp, and sinus ostium opening in patients before and 12 months after surgery. Lund-Mackay system (16) was used to evaluate the sinus condition examined by CT scanning. The total number of points was 24, with 12 points on each side. The recovery of the nasal cavity and paranasal sinus was assessed using the sino-nasal outcome test-20 (SNOT-20).

(6) The subjective olfactory function of patients was determined using T&T olfactometry: five kinds of liquid (perfume, vinegar, ethanol, soy sauce, and sesame oil) were used to detect the olfactory function of patients before and 12 months after surgery. The grades were as follows: euosmia, mild hyposmia, moderate hyposmia, severe hyposmia, and anosmia.

(7) Mucociliary transport rate (MTR): the saccharin test was used to measure the MTR of patients before and 12 months after surgery.

(8) Postoperative complications of patients in the two groups were observed, including nasal bleeding, obstruction, nasal adhesion, and cerebrospinal fluid rhinorrhea.

(9) Quality-of-live scoring: the 36-item short-form (SF-36) rating scale was used to compare the quality-of-life of patients in the two groups before and 12 months after surgery.

### Statistical Analysis

The sample size was calculated by PASS 15.0 (NCSS Statistical Software, Kaysville, Utah). We estimated that a sample of 40 participants in each group was needed for a statistically significant odds ratio (OR) of 2, with an alpha of 0.05 and a beta of 0.2. Based on a 20% drop-out rate, at least 50 cases needed to be included in each group (a total of 100 cases).

All data analyses were conducted using SPSS 21 software (IBM Corp. Armonk, NY, USA). Measurement data were expressed as mean ± *SD* and analyzed by the *t*-test. Enumeration data were expressed as [*n* (%)] and analyzed using the χ^2^ test. A *P* < 0.05 was indicative of a statistically significant difference.

## Results

### Baseline Information

There were 59 patients in the experimental group (32 men and 27 women; mean age: 37.49 ± 6.81 years; mean disease course: 6.65 ± 1.85 years; 39 cases of type I and 20 cases of type II) and 50 patients in the control group (28 men and 22 women; mean age; 38.19 ± 7.06 years; mean disease course: 6.92 ± 2.01 years; 31 cases of type I and 19 cases of type II). There was no significant difference between the two groups with respect to baseline data (all *P* < 0.05) (Table 1).

**Table 1:** Comparison of baseline information of patients between the two groups.

	**Gender (Male/Female, *n*)**	**Age** **(years, x̄ ±s)**	**Disease course (years, x̄ ±s)**	**Chronic rhinosinusitis type (I/II, *n*)**
Control group (*n* = 50)	28/22	38.19 ± 7.06	6.92 ± 2.01	31/19
Experimental group (*n* = 59)	32/27	37.49 ± 6.81	6.65 ± 1.85	39/20
χ^2^/t	0.2886	0.5258	0.7297	0.1982
*P*	0.5911	0.6001	0.4672	0.6562

### Clinical Indices

When compared with the control group, patients in the experimental group had a significantly shorter nasal mucosa recovery time, nasal ventilation time, and a shorter hospital stay (all *P* < 0.05) (Table 2).

**Table 2:** Comparison of clinical indices of patients between the two groups.

	**Intraoperative blood loss (ml)**	**Nasal ventilation time (d)**	**Nasal mucosa recovery time (d)**	**Hospital stay (d)**
Control group (*n* = 50)	104.35 ± 8.26	8.15 ± 1.56	45.61 ± 3.69	7.28 ± 1.06
Experimental group (*n* = 59)	101.54 ± 7.15	6.94 ± 1.18	37.45 ± 3.09	5.66 ± 1.18
χ^2^/t	1.9040	4.6040	12.5668	7.4805
*P*	**0.0596**	**<** **0.0001**	**<** **0.0001**	**<** **0.0001**

*Bold values mean P < 0.05*.

### Therapeutic Effects

There were 38 significantly effective cases, 18 effective cases, and three ineffective cases in the experimental group; in the control group, there were 20 significantly effective cases, 21 effective cases, and nine ineffective cases. Accordingly, the total effective rate for the experimental group (94.9%) was significantly higher than that of the control group (82.0%) (*P* < 0.05) (Table 3).

**Table 3:** Comparison of therapeutic effect of patients between the two groups.

	**Significantly effective**	**Effective**	**Ineffective**	**Total effective rate**
Control group (*n* = 50)	20 (40.0)	21 (42.0)	9 (18.0)	41 (82.0)
Experimental group (*n* = 59)	38 (64.4)	18 (30.5)	3 (5.1)	56 (94.9)
χ^2^/t				4.6081
*P*				**0.0318**

*Bold values mean P < 0.05*.

### Clinical Symptoms

Prior to treatment, there were no significant differences between the two groups in terms of clinical symptoms (*P* > 0.05). After treatment, the scores for both groups had decreased; patients in the experimental group had lower scores than those in the control group (all *P* < 0.05). Besides, the relative improvement rate in the experimental group was significantly greater than in the control group (Table 4).

**Table 4:** Comparison of clinical symptoms of patients between the two groups.

		**Nasal obstruction**	**Running nose**	**Headache**	**Hypogeusia**
Before the treatment	Control group (*n* = 50)	6.15 ± 0.71	6.72 ± 0.88	4.06 ± 0.33	3.97 ± 0.41
	Experimental group (*n* = 59)	6.08 ± 0.64	6.89 ± 0.82	4.11 ± 0.28	3.87 ± 0.37
χ^2^/t		0.5411	1.0429	0.8558	1.3379
*P*		0.5895	0.2993	0.3939	0.1837
After the treatment	Control group (*n* = 50)	1.59 ± 0.20	2.01 ± 0.29	1.55 ± 0.18	1.83 ± 0.19
	Experimental group (*n* = 59)	0.91 ± 0.12	1.44 ± 0.18	1.03 ± 0.10	1.37 ± 0.15
χ^2^/t		21.8873	12.5223	19.0066	14.1190
*P*		**<** **0.0001**	**<** **0.0001**	**<** **0.0001**	**<** **0.0001**
The relative improvement (%)	Control group (*n* = 50)	74.12 ± 5.21	70.12 ± 4.92	61.98 ± 4.36	54.16 ± 2.87
	Experimental group (*n* = 59)	85.08 ± 4.87	78.82 ± 5.05	75.34 ± 5.12	74.59 ± 4.23
χ^2^/t		11.3387	9.0686	14.5192	28.9578
*P*		**<** **0.0001**	**<** **0.0001**	**<** **0.0001**	**<** **0.0001**

*Bold values mean P < 0.05*.

### Inflammatory Cytokines

**Figure 1:**
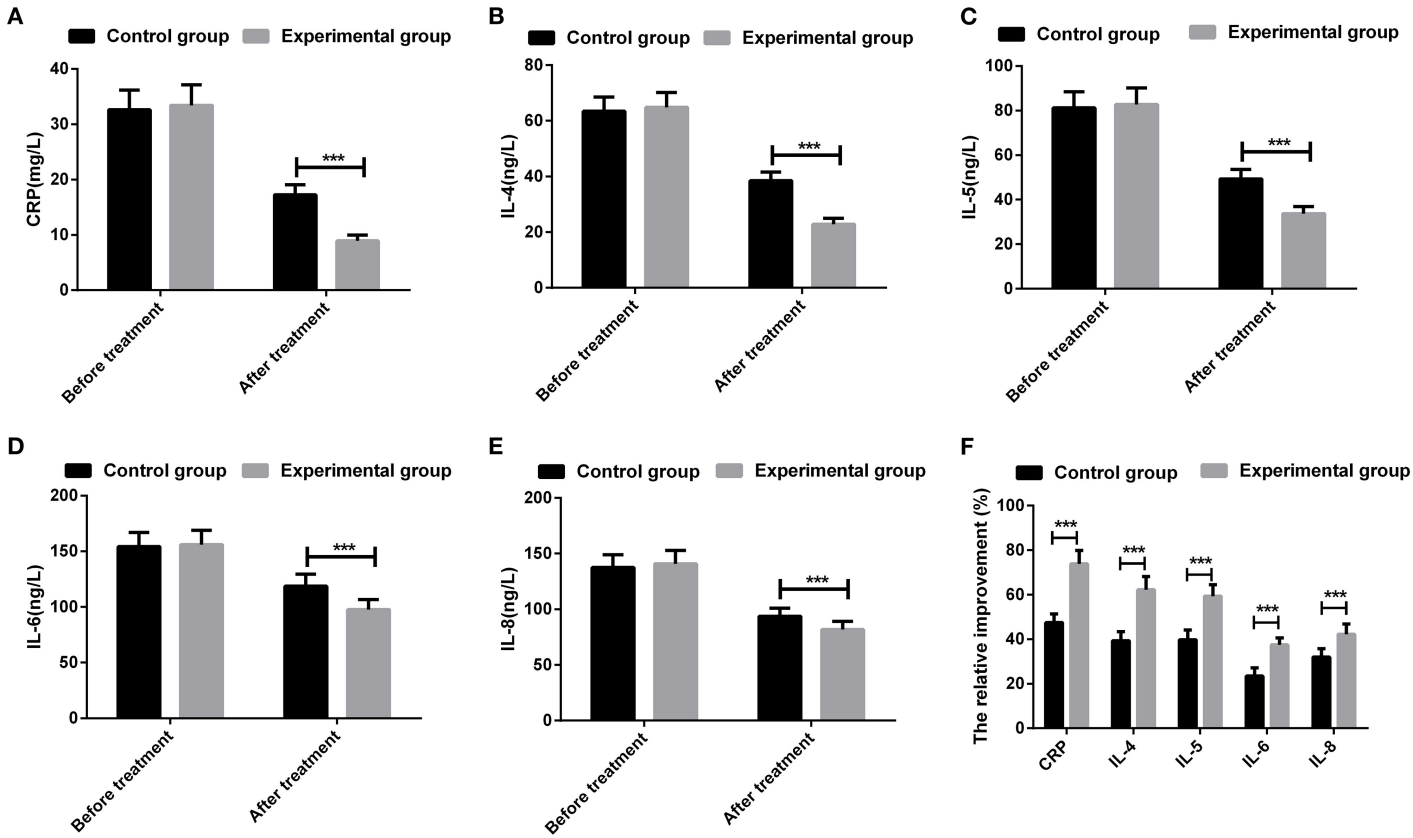
Comparison of inflammatory cytokines of patients between the two groups. **(A)** serum level of C-reactive protein (CRP); **(B)** serum level of interleukin (IL)-4; **(C)** serum level of IL-5; **(D)** serum level of IL-6; **(E)** serum level of IL-8; **(F)** The relative changes of inflammatory cytokines in each group. ***P < 0.001; the measurement data were expressed as mean ± *SD* and analyzed using a *t*-test.

There were no significant differences in the serum levels of CRP, IL-4, IL-5, IL-6, and IL-8 when compared between patients in the two groups prior to treatment (all *P* > 0.05). After treatment, the levels of all inflammatory cytokines were reduced in patients from the two groups; the experimental group had significantly lower levels of CRP, IL-4, IL-5, IL-6, and IL-8 levels than the control group (all *P* < 0.05). In addition, the relative improvement of CRP, IL-4, IL-5, IL-6, and IL-8 differed significantly when compared between the two groups (Figure 1).

### Complications

The total incidence of complications was 8.5% in the experimental group and 22% in the control group; there was a significant difference between the two groups with regard to the incidence of complications (*P* < 0.05) (Table 5).

**Table 5:** Comparison of complications of patients between the two groups.

	**Nasal bleeding**	**Nasal obstruction**	**Nasal adhesion**	**Cerebrospinal fluid rhinorrhea**	**Total complication incidence**
Control group (*n* = 50)	3 (6.0)	3 (6.0)	4 (8.0)	1 (2.0)	11 (22.0)
Experimental group (*n* = 59)	1 (1.7)	1 (1.7)	2 (3.4)	1 (1.7)	5 (8.5)
χ^2^/t					3.9531
*P*					**0.0468**

*Bold values mean P < 0.05*.

### Recovery of the Nasal Mucosa

Prior to surgery, nasal endoscopy showed that the olfactory cleft was narrowed, the olfactory mucosa was pale and there was local purulent secreta retention in patients from both groups. Four months after surgery, epithelization was observed in the operative cavities; there was no secreta in the middle nasal meatus, the color of the olfactory mucosa was gradually restored, and there was no swelling in the mucosa and no retention of secreta. Six months after surgery, we determined Lund-Mackay and SNOT-20 scores to evaluate the recovery of the nasal mucosa. We found that patients in the experimental group had significantly lower Lund-Mackay and SNOT-20 scores than those in the control group (*P* < 0.05), thus indicating a better nasal mucosal recovery in the experimental group (Table 6).

**Table 6:** Comparison of nasal mucosal recovery of patients between the two groups.

	**Lund-Mackay score**	**SNOT-20 score**
Control group (*n* = 50)	2.18 ± 0.41	5.34 ± 0.52
Experimental group (*n* = 59)	1.83 ± 0.30	4.11 ± 0.46
χ^2^/t	5.1343	13.1019
*P*	**<** **0.0001**	**<** **0.0001**

*SNOT-20, sino-nasal outcome test-20. Bold values mean P < 0.05*.

### Olfactory Function and MTR

Prior to surgery, there was no significant difference between the two groups with regard to olfactory function and MTR (both *P* > 0.05). After surgery, patients in the experimental group showed significantly improved olfactory function and significantly increased MTR than those in the control group (both *P* < 0.05) (Table 7).

**Table 7:** Comparison of olfactory function and MTR of patients between the two groups.

		**Olfactory function**				**MTR (x ±s, mm/min)**
		**Euosmia**	**Mild hyposmia**	**Moderate hyposmia**	**Severe hyposmia**	
Before the surgery	Control group (*n* = 50)	0	18 (36.0)	21 (42.0)	11 (22.0)	4.16 ± 0.48
	Experimental group (*n* = 59)	0	23 (39.0)	26 (44.1)	10 (16.9)	4.03 ± 0.53
χ^2^/t		0.4492				1.3321
*P*		0.7988				0.1856
Six months after the surgery	Control group (*n* = 50)	22 (44.0)	6 (12.0)	13 (26.0)	9 (18.0)	5.91 ± 0.66
	Experimental group (*n* = 59)	37 (62.7)	11 (18.6)	7 (11.9)	4 (6.8)	7.73 ± 0.81
χ^2^/t		8.3213				12.7079
*P*		**0.0398**				**<** **0.0001**

*MTR, mucociliary transport rate. Bold values mean P < 0.05*.

### Quality-of-Life

Next, we determined the quality-of-life of patients in the two groups 12 months after surgery. We found that there was no significant difference between the two groups with regards to mental health, role-physical, physiological function, emotional character, social function, and pain (all *P* > 0.05), thus indicating that 12 months after surgery, patients in the two groups had a similar quality-of-life (Table 8).

**Table 8:** Comparison of quality of life of patients between the two groups.

	**Metal health**	**Role-physical**	**Physiological function**	**Emotional character**	**Social function**	**Pain**
Control group (*n* = 50)	85.62 ± 7.26	80.61 ± 7.05	82.15 ± 6.25	72.05 ± 4.47	82.87 ± 8.26	79.25 ± 6.25
Experimental group (*n* = 59)	87.91 ± 7.82	82.37 ± 8.15	84.68 ± 7.64	73.84 ± 5.97	85.71 ± 9.81	81.26 ± 7.48
χ^2^/t	1.5740	1.1944	1.8702	1.7453	1.6177	1.5059
*P*	0.1184	0.2349	0.0642	0.0838	0.1087	0.1350

## Discussion

In this study, we explored the efficacy of FESS combined with TAA AQ for the treatment of CRS. Based on comprehensive comparisons of clinical indices, therapeutic effects, clinical symptoms, inflammatory cytokines, complications, nasal mucosal recovery, olfactory function, MTR, and quality-of-life, we found that compared with FESS alone, the combination of FESS and TAA AQ had a better therapeutic effect on patients with CRS.

Functional endoscopic sinus surgery was tailored to disease extent and focused on restoring mucociliary ventilation and clearance. Thus, FESS is considered the standard surgical approach for CRS which does not respond to medical treatment. The inflammatory process in patients with CRS after FESS plays an essential role in recurrence and wound healing and, thus, the continued use of postoperative medical therapy (such as topical or oral corticosteroids, saline irrigation, and antibiotics) is recommended for CSR patients after FESS to facilitate a successful outcome (17). Nasal steroids are the safest, most effective, and most potent medications available for treating inflammation in the nasal and sinus mucosa. Improvement of the generally poor adherence rates of the intranasal steroid treatment of CRS after FESS is vital if we are to achieve adequate control of symptoms, good wound healing, and minimal chance of relapse. In a previous study, Zhang *et al*. investigated the efficacy and potential of self-crosslinked hyaluronic acid (scHA) gel as a topical drug sustained-release carrier for the steroid budesonide in CRSwNP patients who underwent FESS. These authors found that scHA/budesonide is a valuable treatment for FESS postoperative management (18). A recent systematic review, featuring eight studies, identified four studies that applied triamcinolone as the operative steroid; analysis found that triamcinolone, as an operative steroid-impregnated nasal packing, appears to exert positive effects on postoperative endoscopic outcomes in CRSwNP patients undergoing FESS (11). Vaidyanathan *et al*. previously designed a randomized clinical trial involving 60 subjects with CRS and found that an initial dose of oral corticosteroids followed by topical treatment significantly improved olfactory function when compared to topical therapy alone (19). In addition, another study which included 60 patients with CRS who received FESS, and those treated with TAA AQ and normal saline, revealed that they experienced augmentation of smell function throughout the study, while patients with CRS who received triamcinolone showed better improvement after 8 weeks when compared with those treated with normal saline (13). These data indicate the potential of TAA AQ for wound healing and the improvement of olfactory function; however, the specific clinical condition of the patients was not considered in sufficient detail.

To further evaluate the efficacy of FESS combined with TAA AQ, we classified 109 patients with CRS into a control group and an experimental group and compared the clinical indices, total effective rate, clinical symptoms, pre- and postoperative serum levels of inflammatory cytokines, the recovery of the nasal mucosa, olfactory function, MTR, postoperative complications, and the quality- of-life of patients between the two groups. Analysis showed that when compared to patients with CRS treated with FESS alone, those treated with FESS and TAA AQ had improved clinical indices and olfactory function, better therapeutic effects, enhanced nasal mucosal recovery, and reduced clinical symptoms, inflammatory cytokine levels, and complications. There were no significant differences in the quality-of-life of patients when compared between the two groups. These data revealed a better therapeutic effect of FESS when combined with TAA AQ in patients with CRS.

In this study, we further confirmed the superiority of FESS and TAA AQ for the treatment of patients with CRS, thus providing a basis for the medical management of patients with CRS after FESS. However, as the number of studies involved in this analysis was not sufficient, our conclusions may not be robust. Furthermore, the small sample size and the lack of comparison between TAA AQ and other medical treatments represented significant limitations of the present study. Future studies, with larger sample sizes, are now required to increase the rigor of our findings.

In conclusion, this study demonstrated that when compared with FESS alone, FESS combined with TAA AQ had a better therapeutic effect on patients with CRS, with slight inflammation, reduced complications, improved clinical symptoms, and improved olfactory function. However, the rate of cerebrospinal fluid leakage indicated that we must consider the safety profile and bioavailability of triamcinolone acetonide before long-term use (20). More evidence and data are now needed to further confirm our findings using more comprehensive and well-designed studies.

## Data Availability Statement

The original contributions presented in the study are included in the article/supplementary material, further inquiries can be directed to the corresponding author.

## Author Contributions

ZH finished study design and manuscript editing. ZH and HG finished experimental studies. ZH and WL finished data analysis. All authors have read and approved the final manuscript.

## Conflict of Interest

The authors declare that the research was conducted in the absence of any commercial or financial relationships that could be construed as a potential conflict of interest.

## Publisher's Note

All claims expressed in this article are solely those of the authors and do not necessarily represent those of their affiliated organizations, or those of the publisher, the editors and the reviewers. Any product that may be evaluated in this article, or claim that may be made by its manufacturer, is not guaranteed or endorsed by the publisher.
